# Supplementary material for: Splice-Junction-Based Mapping of Alternative Isoforms in the Human Proteome
Source: Cell Rep. Author manuscript; Available in PMC 2020 Jan 15. (PMC6961840; doi:10.1016/j.celrep.2019.11.026)
Supplement: 3 [file NIHMS1546469-supplement-3.zip › DF2/PXD006675/PulmonaryArtery-92-Q13332-SGALQIESSEETDQGK.pdf]

A

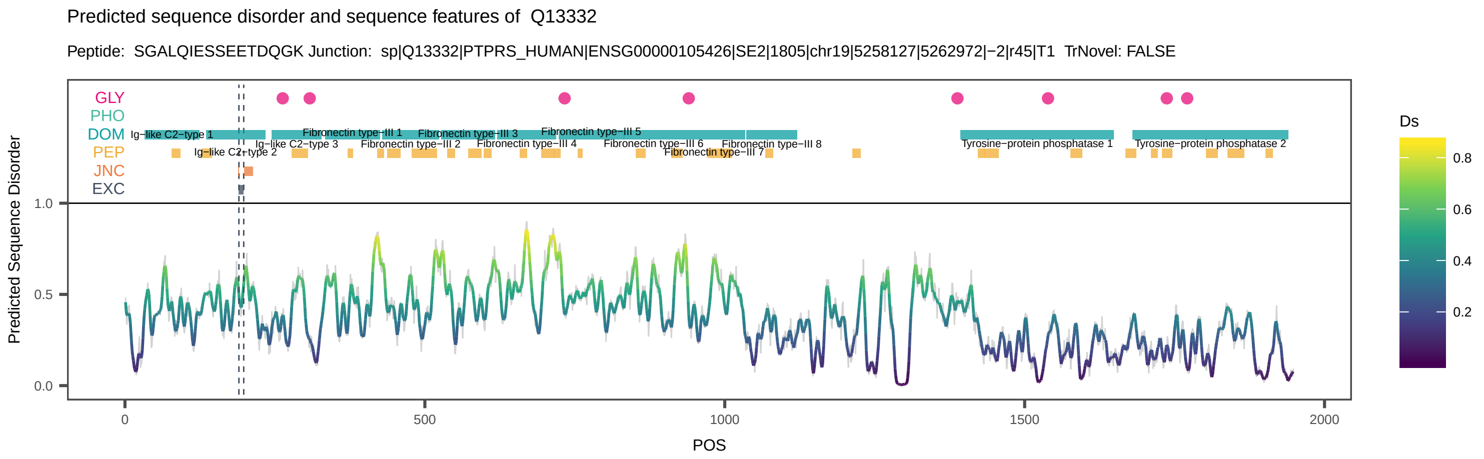
Predicted sequence disorder and sequence features of Q13332
Peptide: SGALQIESSEETDQGK Junction: sp|Q13332|PTPRS_HUMAN|ENSG00000105426|SE2|1805|chr19|5258127|5262972|−2|r45|T1 TrNovel: FALSE
GLY
PHO
DOM
PEP
JNC
EXC
Ig−like C2−type 1
Ig−like C2−type 2
Ig−like C2−type 3
Fibronectin type−III 1
Fibronectin type−III 2
Fibronectin type−III 3
Fibronectin type−III 4
Fibronectin type−III 5
Fibronectin type−III 6
Fibronectin type−III 7
Fibronectin type−III 8
Tyrosine−protein phosphatase 1
Tyrosine−protein phosphatase 2
Predicted Sequence Disorder
1.0
0.5
0.0
0
500
1000
1500
2000
POS
Ds
0.8
0.6
0.4
0.2

B

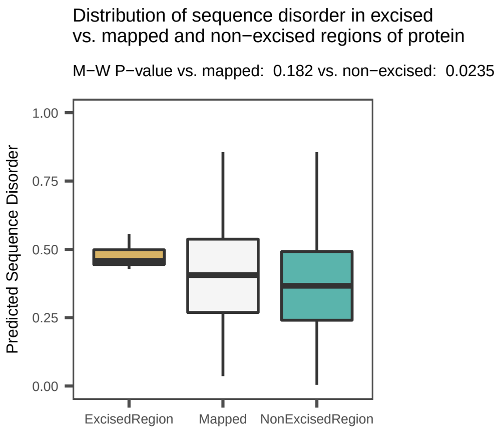
Distribution of sequence disorder in excised
vs. mapped and non−excised regions of protein
M−W P−value vs. mapped: 0.182 vs. non−excised: 0.0235
Predicted Sequence Disorder
1.00
0.75
0.50
0.25
0.00
ExcisedRegion
Mapped
NonExcisedRegion

C
